# Accumulating evidence suggests that some waterbird species are potential vectors of *Vibrio cholerae*

**DOI:** 10.1371/journal.ppat.1007814

**Published:** 2019-08-22

**Authors:** Sivan Laviad-Shitrit, Ido Izhaki, Malka Halpern

**Affiliations:** 1 Department of Evolutionary and Environmental Biology, Faculty of Natural Sciences, University of Haifa, Haifa, Israel; 2 Department of Biology and Environment, Faculty of Natural Sciences, University of Haifa, Oranim, Tivon, Israel; Geisel School of Medicine at Dartmouth, UNITED STATES

## Abstract

*Vibrio cholerae* is the causative agent of cholera, a life-threatening diarrheal disease. Cholera causes epidemics and pandemics, but the ways this disease spreads worldwide is still unclear. This review highlights a relatively new hypothesis regarding the way *V*. *cholerae* can be globally dispersed. Copepods and chironomids are natural reservoirs of *V*. *cholerae* and are part of different fish species’ diet. Furthermore, *V*. *cholerae* inhabits marine and freshwater fish species. Waterbird species feed on fish or on small invertebrates such as copepods and chironomids. Waterbirds have also been found to carry living copepods and/or chironomids internally or externally from one waterbody to another. All of the above points to the fact that some waterbird species might be vectors of *V*. *cholerae*. Indeed, we and others have found evidence for the presence of *V*. *cholerae* non-O1 as well as O1 in waterbird cloacal swabs, feces, and intestine samples. Moreover, hand-reared cormorants that were fed on tilapia, a fish that naturally carries *V*. *cholerae*, became infected with this bacterial species, demonstrating that *V*. *cholerae* can be transferred to cormorants from their fish prey. Great cormorants as well as other waterbird species can cover distances of up to 1,000 km/day and thus may potentially transfer *V*. *cholerae* in a short time across and between continents. We hope this review will inspire further studies regarding the understanding of the waterbirds' role in the global dissemination of *V*. *cholerae*.

## Introduction

Birds are ubiquitous and globally distributed. There are 10,000 known bird species, which account for over 15% of all vertebrates [1]. Waterbirds are birds that live on or around fresh water or marine water. Some waterbirds dive from the surface or the air to catch prey in water, and others have legs adapted to feed in water. Most studies on birds' bacterial communities have been conducted on poultry or terrestrial birds, usually by sampling feces or swab samples [2,3]. Only a few studies have been conducted on wild waterbird microbiomes [4–11]. Billions of wild waterbirds migrate between continents twice a year in a period of only a few weeks [12]. These wild waterbirds may have a role in pathogen (e.g., bacteria, archaea, fungi, viruses, protozoa) dissemination and are extremely important in respect to public health [12]. Here, we review current knowledge on the topic of a relatively new hypothesis that has been presented by Halpern and colleagues [13], positing that waterbirds might be vectors of *Vibrio cholerae*, and thus may distribute this species all over the globe.

### V. cholerae

*V*. *cholerae* is a gram-negative, facultative anaerobe, motile curved rod. It belongs to the family of *Vibrionaceae* and is the etiological agent of cholera, a life-threatening disease. Strains belonging to *V*. *cholerae* inhabit both marine and freshwater ecosystems [14]. There are more than 200 *V*. *cholerae* serogroups, but only serogroups O1 and O139 have been associated with cholera endemics and pandemics [15]. *V*. *cholerae* non-O1/O139 strains can also cause intestinal and extra-intestinal infections such as gastroenteritis, cholera-like diarrhea, wound infections, external otitis, and bacteremia that sometimes can be fatal in humans [16–18].

### Cholera

Cholera is a severe diarrheal disease that has afflicted human beings and shaped human history for over 2 millennia [15,18]. The disease spreads throughout and between continents causing epidemics and pandemics and kills thousands of people annually. Humans can become infected with *V*. *cholerae* serogroups O1 or O139 by consuming contaminated food or water. Toxigenic strains cross the human gastric acid barrier and then colonize the small intestine epithelial cells. After colonization, the bacterium produces the cholera toxin, which triggers fluid secretion by the intestinal epithelium, causing acute dehydration [15,19]. WHO evaluated that about 3 million people are exposed to cholera every year, and this leads to 95,000 deaths annually [15].

An example of a cholera epidemic is the outbreak in Haiti in October 2010. Diverse studies pointed out that the clinical isolates from that event were most closely related to Asian isolates [20,21]. Studies that compared the whole genome sequences of *V*. *cholerae* strains from different geographic regions suggested that the bacterium was introduced into Haiti from Nepal by humans [22–24]. Since April 2017, there has been a cholera epidemic in Yemen with 1,207,596 suspected cases and 2,510 associated deaths [25]. Weill and colleagues [26] compared the whole genome sequences of *V*. *cholerae* strains from the Yemen epidemic with strains from Asia and Africa. They concluded that the source of the epidemic strains in Yemen is a strain related to a cholera outbreak in South Asia first detected in 2012. However, this strain, which entered Yemen in 2016, had been circulating and causing outbreaks in eastern Africa in 2013 through 2014 before it appeared in Yemen in 2016 [26].

### Potential reservoirs of *V*. *cholerae*

*V*. *cholerae* is part of the normal microbial population and ecology of the surface water of our planet. Colwell and colleages [27–30] showed that *V*. *cholerae* proliferates while attached to or associated with eukaryotic organisms in the aquatic environment, particularly copepods (*Crustacea*). We found indications that chironomids serve as reservoirs for *V*. *cholerae* [31–38]. Chironomids (*Diptera*) are one of the most widely distributed insects in marine and freshwater habitats. *V*. *cholerae* was isolated from all 4 life stages of chironomids [37]. It has been demonstrated that *V*. *cholerae* can survive better in seawater when it is associated to zooplankton than as a free cell [27].

In laboratory studies, serogroups O1 and O139 were able to grow and survive in the cytoplasm of trophozoites and in the cysts of free-living amoeba *Acanthamoeba castellanii* [39, 40]. Arthropods [41], oysters [42], cyanobacteria, diatoms, and phaeophytes [43] were also suggested as carriers of *V*. *cholerae*.

### Fish and *V*. *cholerae*

Senderovich and colleagues [44] surveyed for the first time the presence of *V*. *cholerae* in fish intestines in various water habitats in Israel. They found 11 fish species that inhabited *V*. *cholerae* (10 species from fresh water habitats and one from a marine habitat). One species (tilapia) harbored 5 × 10^3^ colony-forming units (cfu) per 1 g intestinal content. They suggested that fish can be a reservoir of *V*. *cholerae* and can act as small-scale vectors for the dispersal of this bacterium [44]. Fish consume copepods and chironomids, so these food items might be the source of *V*. *cholerae* in the fish gut. Correlation of some cholera outbreaks with the consumption of uncooked fish has been reported [45–48]. Senderovich and colleagues [44] suggested that in the fish intestines *V*. *cholerae* may have a role in chitin degradation. Thus, the fish host and *V*. *cholerae* may have a commensal relationships [44].

Halpern and Izhaki [49] reviewed the literature on fish as reservoirs for *V*. *cholerae*. *V*. *cholerae* was isolated from fish intestines, gills, skin, kidney, liver, and brain tissues, and in total, were identified in 30 fish species [49]. In most cases, the fish were healthy. Runft and colleagues [50] infected naive zebrafish with *V*. *cholerae* O1 and showed that the bacteria could attach to a fish’s intestinal epithelium and form microcolonies. They also showed that contaminated fish could spread the bacterium to naive fish.

Recently, more evidence on the presence of *V*. *cholerae* in fish has accumulated. For example, Hossain and colleagues [51] studied the potential of Hilsha fish (*Tenualosa ilisha*) to act as a vector of *V*. *cholerae* to humans. This fish migrates from cholera-endemic areas to freshwater rivers around Bangladesh and is the most consumed fish species in that country. They found that about 16% of their isolates (*n* = 158) were *V*. *cholerae* O1 strains [51]. *V*. *cholerae* O1 was also isolated from the gills of a freshwater fish [52] and from a Chinese freshwater fish [53]. Fifty-three *V*. *cholerae* non-O1/O139 isolates were identified from Malaysian fish [54] and from ornamental fish originating in south-east Asian countries [55].

### *V*. *cholerae* dissemination

Cholera spreads all over the globe and causes epidemics and pandemics. Nevertheless, despite intensive research efforts, its ecology remains an enigma, in particular the mechanism that enables *V*. *cholerae* to cross water bodies and even oceans. Huq and Colwell [56] suggested that *V*. *cholerae* cells are dispersed in the water while attached to copepods and this serves as a mechanism for its global distribution. However, humans consume freshwater whereas the copepods’ journey between continents occurs in the ocean (marine water). Broza and colleagues [31] suggested that flying chironomid adults may disseminate the bacterium between water bodies; however, this dissemination is restricted to short distances.

**Fig 1:**
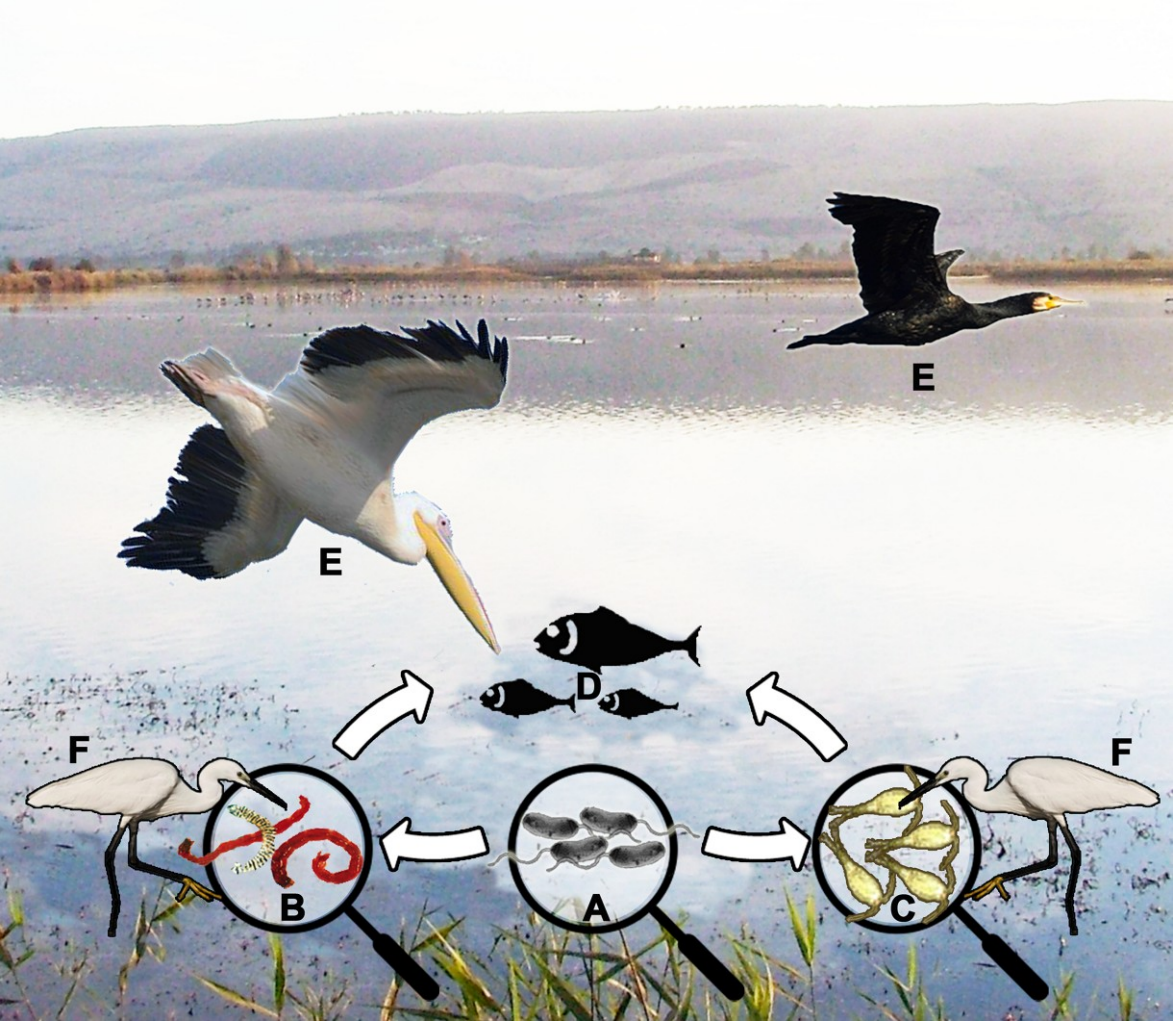
A diagram demonstarting possible ways of *V*. *cholerae* global dissemination. *V*. *cholerae* (A) can be transmitted from its natural reserviors by chironomids (B) and/or copepods (C) via fish (D) to different species of waterbirds (E) or directly from the zooplankton (B or C) to waterbird species (F).

Many waterbird species move within and between marine and fresh waters (e.g., pelicans, cormorants, gulls) [57–59]. Therefore, Halpern and colleagues [13] hypothesized that migratory waterbirds may disseminate *V*. *cholerae* within and between continents. They suggested that the bacterium can pass from endemic to uninfected water bodies via waterbirds in 2 courses: (i) waterbirds may carry directly contaminated copepods and/or chironomids (Fig 1); (ii) waterbirds may consume fish that feed on copepods or chironomids [13,60] (Fig 1). Their hypothesis was based on the findings of Green and Sanchez [61] and of Frisch and colleagues [62] that chironomids and copepods can survive the gut passage of several waterbird species or can become externally attached to birds' feet and feathers. Consequently, dispersal of these invertebrates via waterbirds may be a common phenomenon and an important process for *V*. *cholerae* dispersion (Fig 1).

Halpern and colleagues [13] also found in the literature studies reporting the presence of *V*. *cholerae* in waterbirds. These studies had become overlooked and forgotten over the years. Bisgaard and Kristensen [63] isolated *V*. *cholerae* from 2 ducklings at a Danish duck farm (Table 1). Lee and colleagues [64] isolated *V*. *cholerae* in Kent, England, from cloacal swabs of black-headed gulls (*Larus ridibundus*), great black-backed gulls (*Larus marinus*), herring gulls (*Larus argentatus*), and rooks (*Corvus frugilegus*). In 1980, *V*. *cholerae* was isolated from the liver and heart of a domestic goose (*Anser anser*) [65] (Table 1). In a study that was performed in Utah and Colorado, *V*. *cholerae* was isolated from cloacal swabs and fresh droppings that were collected from 20 waterbird species [66]. *V*. *cholerae* non-O1/O139 were detected in coots, cormorants, gadwalls, gulls, herons, killdeers, mallards, pelicans, pintails, teals, redheads, egrets, ibises, and phalaropes species [66] (Table 1). The non-O1/O139 isolates (*n* = approximately 200) were classified into 22 different serogroups (O11, O12, O14, O16, O17, O19, O22, O23, O31, O43, O44, O48, O60, O102, O106, O148, O176, O312, O340, O355, O359, O360, and some were not identified to their serogroup) [66] (Table 1). Moreover, in the same study, *V*. *cholerae* serogroup O1 biotype El Tor Ogawa was isolated from cloacal swabs and fresh feces of the great blue heron (*Ardea herodias*) and ring-billed gull (*Larus delawarensis*). Unstable O1 serogroup was detected from 3 other waterbird species: California gull (*Larus californicus*), American coot (*Fulica Americana*), and a double-crested cormorant (*Phalacrocorax auritus*). This means that out of the 20 studied waterbird species, 5 (25%) carried pathogenic serogroups [66] (Table 1). *V*. *cholerae* non-O1 was also identified from gulls that were sampled in Connecticut [67].

**Table 1:** Isolation of *V*. *cholerae* strains from waterbird species sampled from different regions around the world (data from studies published between 1975 and 2018).

Bird species	Country of isolation	Isolation source	Non-O1/O139	O1	Reference
*Anas* spp. (duck)	Denmark	Conjunctiva and intestines	+	−	[63]
*Anas acuta* (northern pintail)	Colorado and Utah	Cloacal swab, fresh feces	+	−	[66]
*Anas carolinensis* (green-winged teal)	Colorado and Utah	Cloacal swab, fresh feces	+	−	[66]
*Anas cyanoptera* (cinnamon teal)	Colorado and Utah	Cloacal swab, fresh feces	+	−	[66]
*Spatula discors* (blue-winged teal)	Colorado and Utah	Cloacal swab, fresh feces	+	−	[66]
*Anas platyrhynchos* (mallard)	Colorado and Utah	Cloacal swab, fresh feces	+	−	[66]
*Mareca strepera* (gadwall)	Colorado and Utah	Cloacal swab, fresh feces	+	−	[66]
*Anser anser* (greylag goose)	Florida	Liver and heart	+	−	[65]
*Ardea herodias* (great blue heron)	Colorado and Utah	Cloacal swab, fresh feces	+	+	[66]
*Aythya americana* (redhead)	Colorado and Utah	Cloacal swab, fresh feces	+	−	[66]
*Bubulcus ibis* (cattle egret)	Colorado and Utah	Cloacal swab, fresh feces	+	−	[66]
*Calidris* spp. (sandpipers)	Venezuela	Fecal samples	+	−	[69]
*Calidris pusilla* (semipalmated sandpiper)	Venezuela	Fecal samples	+	−	[69]
*Charadrius wilsonia* (Wilson's plover)	Venezuela	Fecal samples	+	−	[68]
*Charadrius vociferus* (killdeer)	Colorado and Utah,	Cloacal swab, fresh feces	+	−	[66]
*Chroicocephalus cirrocephalus* (grey-headed gull)	Brazil	Cloacal swab	+	−	[71]
*Corvus frugilegus* (rook)	England	Cloacal swab	+	−	[64]
*Egretta garzetta* (little egret)	Israel	Intestine	+	+*	[72]
*Egretta thula* (snowy egret)	Colorado and Utah	Cloacal swab, fresh feces	+	−	[66]
*Fulica americana* (American coot)	Colorado and Utah	Cloacal swab, fresh feces	+	+	[66]
*Jacana jacana* (wattled jacana)	Venezuela	Fecal samples	+	−	[69]
*Larus* spp. (gulls)	Connecticut	fresh feces	+	−	[67]
*Larus argentatus* (European herring gull)	England	Cloacal swab	+	−	[64]
*Larus californicus* (California gull)	Colorado and Utah	Cloacal swab, fresh feces	+	−	[66]
*Larus delawarensis* (ring-billed gull)	Colorado and Utah	Cloacal swab, fresh feces	+	+	[66]
*Larus dominicanus* (kelp gull)	Brazil	Cloacal swab	+	−	[71]
*Larus marinus* (great black-backed gull)	England	Cloacal swab	+	−	[64]
*Larus ridibundus* (black-headed gull)	England; Israel	Cloacal swab, intestine	+*	−	[64,72]
*Leucophaeus pipixcan* (Franklin's gull)	Colorado and Utah	Cloacal swab, fresh feces	+	−	[66]
*Nycticorax nycticorax* (black-crowned night heron)	Colorado and Utah; Israel	Cloacal swab, fresh feces, intestine	+	+*	[66,72]
*Pelecanus erythrorhynchos* (American white pelican)	Colorado and Utah	Cloacal swab, fresh feces	+	−	[66]
*Phaetusa simplex* (large-billed tern)	Venezuela	Fecal samples	+	−	[69]
*Phalacrocorax auritus* (double-crested cormorant)	Colorado and Utah	Cloacal swab, fresh feces	+	+	[66]
*Phalacrocorax carbo* (great cormorant)	Israel	Intestine	+	+*	[8]
*Phalaropus tricolor* (Wilson's phalarope)	Colorado and Utah	Cloacal swab, fresh feces	+	−	[66]
*Phoenicopterus ruber* (American flamingo)	Venezuela	Fecal samples	+	−	[69]
*Plegadis chihi* (white-faced ibis)	Colorado and Utah	Cloacal swab, fresh feces	+	−	[66]
*Puffinus puffinus* (Manx shearwater)	Rio de Janeiro, Brazil	Cloaca, oral, ocular, and tracheal swabs	+	−	[70]
*Sula leucogaster* (brown booby)	Brazil	Cloacal swab	+	−	[71]
*Thalassarche chlororhynchos* (Atlantic yellow-nosed albatross)	Brazil	Cloacal swab	+	−	[71]
*Thalasseus acuflavidus* (Cabot’s tern)	Brazil	Cloacal swab	+	−	[71]
*Tringa melanoleuca* (greater yellowlegs)	Venezuela	Fecal samples	−	+	[68]

*Detected by molecular methods.

All of these findings from relatively old published literature (before 1989) regarding *V*. *cholerae* isolation from different waterbird species strongly support the hypothesis that migratory waterbirds may serve as vectors for *V*. *cholerae* [13,60].

### Waterbirds and *V*. *cholerae*

Following the hypothesis that migratory waterbirds may disseminate *V*. *cholerae* [13,60], more studies regarding the presence of *V*. *cholerae* in waterbirds have been reported. In Venezuela, *V*. *cholerae* O1 Inaba El Tor and *V*. *cholerae* non-O1 were isolated and identified from 6 greater yellowlegs (*Tringa melanoleuca*) and from 6 Wilson's plover (*Charadrius wilsonia*), respectively [68]. Fernández-Delgado and colleagues [69] studied the prevalence of *Vibrio* spp. in fecal samples of resident and migratory waterbirds around 2 costal sites in the tropical southern Caribbean Sea, Venezuela. They isolated *V*. *cholerae* from 5 waterbird species: sandpipers (*Calidris* spp.), large-billed tern (*Phaetusa simplex*), American flamingo (*Phoenicopterus ruber*), wattled jacana (*Jacana jacana*), and semipalmated sandpiper (*Calidris pusilla*) [69] (Table 1). *V*. *cholerae* non-O1/non-O139 was isolated from cloacal swab samples of wild manx shearwater (*Puffinus puffinus*) that were caught in the north-central coast of Rio de Janeiro, Brazil [70]. In another survey that was conducted in the same place a few years later, Cardoso and colleagues [71] isolated and identified *V*. *cholerae* non-O1/non-O139 isolates from waterbird species: kelp gull (*Larus dominicanus*), Atlantic yellow-nosed albatross (*Thalassarche chlororhynchos*), brown booby (*Sula leucogaster*), Cabot’s tern (*Thalasseus acuflavidus*), and grey-headed gull (*Chroicocephalus cirrocephalus*) (Table 1, S1 Table).

**Fig 2:**
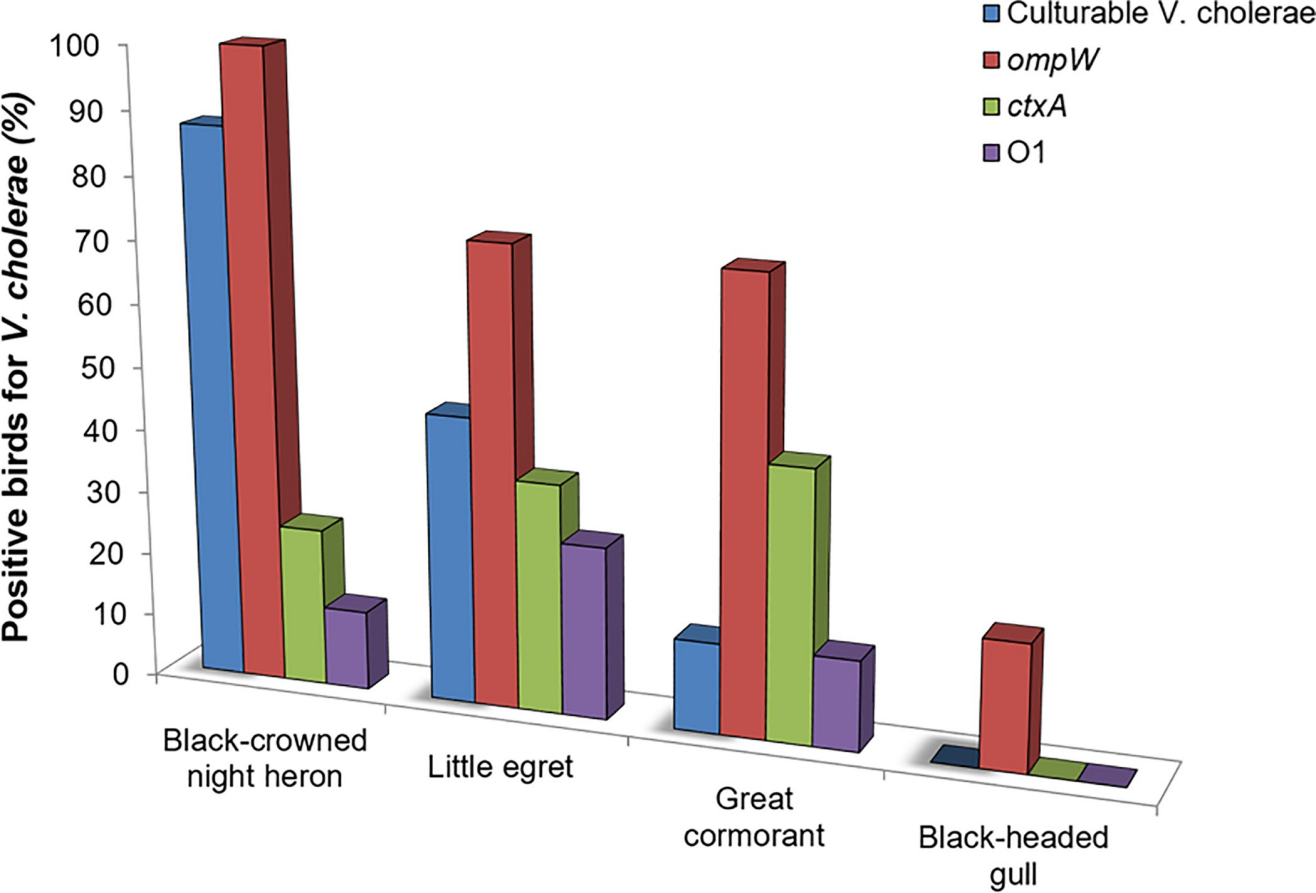
The existence of culturable and unculturable (detected by PCR amplification of *OmpW* gene) *V*. *cholerae* in 4 wild waterbird species intestine samples. In addition, evidence for the presence of *V*. *cholerae* O1 serogroup and cholera toxin were also detected using specific PCR amplifications (data from Laviad-Shitrit and colleagues [8,72]).

Laviad-Shitrit and colleagues [8] isolated *V*. *cholerae* non-O1/O139 from the intestines of 1 out of 7 wild great cormorant (*Phalacrocorax carbo*) individuals sampled in Israel. They detected by molecular tools the presence of *ompW* gene in 5 individual cormorants, demonstrating the presence of *V*. *cholerae* in 5 out of 7 wild cormorants. The presence of cholera toxin subunit A (*ctxA*) and serogroup O1 was also molecularly detected in the intestine of 3 and 1 individual cormorants, respectively (Table 1, Fig 2). In another study, *V*. *cholerae* was detected in the intestine of 3 wild waterbird species in Israel: little egret (*Egretta garzetta*), black-crowned night heron (*Nycticorax nycticorax*), and black-headed gull (*Larus ridibundus*). Forty-six *V*. *cholerae* isolates were obtained from the intestines of little egrets and black-crowned night herons. These isolates were classified into 23 different serogroups (O6, O8, O9, O13, O16, O18, O21, O33, O36, O39, O40, O65, O85, O93, O94, O103, O123, O125, O126, O128, O171, O193, and O195). All isolates were found positive for *toxR* gene and negative for *ctxA*, *tcpA*, *tcpI*, *zot*, and *ace* genes. In addition, *hapA* was found in 95.3% of the isolates, *hlyA* in 93.0%, *ompU* in 41.0%, and 9.7% were found positive for some of the type three secretion system (TTSS) genes (*vcsC2*, *vcsN2*, *vspD* and *vcsV2*) [72]. More than one serogroup was identified from the same intestinal sample, suggesting that different *V*. *cholerae* serogroups inhabit the intestine of an individual. Although *V*. *cholerae* was not isolated from black-headed gulls, the presence of *ompW* gene, which identifies the presence of *V*. *cholerae*, was detected in 1 out of 5 black-headed gull intestine samples, indicating that *V*. *cholerae* was present in this bird species (Table 1. Fig 2). Interestingly, the genes for serogroup O1 and cholera toxin were detected in some of the waterbird intestine samples of little egrets and black-crowned night herons [72] (Table 1. Fig 2).

### Fish, waterbirds, and *V*. *cholerae*

Laviad-Shitrit and colleagues [8] studied whether waterbirds that fed on fish could act as vectors for *V*. *cholerae* by consuming naturally colonized fish. They tested 8 captive hand-reared great cormorants, divided into 2 groups: (i) the control group, which fed on golden fish that was negative to the presence of *V*. *cholerae* and (ii) the experimental group that fed on tilapia that is naturally colonized by *V*. *cholerae*. In the control group, both the fish and the cormorants were negative to *V*. *cholerae* throughout the 3 weeks of the experiment. In the experimental group, *V*. *cholerae* was transferred from the naturally colonized tilapia to the cormorants and was detected in the cormorants’ feces. They also demonstrated that *V*. *cholerae* could survive in the cormorants’ digestive tract even 72 hours after tilapia ingestion. According to the literature, in a period of 72 hours, great cormorants are able to cross oceans [8].

### Antimicrobial resistance in *V*. *cholerae* isolates from birds

Laviad-Shitrit and colleagues [73] studied the antimicrobial susceptibilities of environmental *V*. *cholerae* strains isolated from waterbird intestine samples. They found that waterbirds showed the highest minimal inhibitory concentration (MIC) values to all studied antimicrobial agents (except ampicillin) compared with strains isolated from fish or chironomids [73]. Cardoso and colleagues [71] isolated *V*. *cholerae* resistant to ampicillin from the brown booby (*Sula leucogaster*). Hence, waterbirds may also be vectors for antimicrobial resistant strains and may spread them globally. It is the responsibility of local administrations to monitor areas with large migratory waterbird populations for *V*. *cholerae* presence and for antimicrobial resistance properties of the bacteria.

## Conclusions

Local or intercontinental migratory movements of waterbirds and fish provide a possible mechanism for the introduction of new endemic foci of disease at short or great distances from the original source of *V*. *cholerae* infection (Fig 1). Therefore, we advocate that future studies on the occurrence of cholera outbreaks, especially across remote geographical regions, should consider the possible role of waterbirds and fish in *V*. *cholerae* transmission locally or globally. Epidemiological studies should examine the connection between environmental *V*. *cholerae* strains from waterbirds and fish to cholera cases. A fuller understanding of the ecology of *V*. *cholerae* is of vital interest to help limit the times that humans come into contact with this pathogen.

Furthermore, to prove that *V*. *cholerae* specific strains are disseminated by waterbirds from one location to another, a comparative genetic analysis of *V*. *cholerae* strains from distinct locations should be performed. To this end, waterbirds and water bodies should be sampled at different periods of the year that coincide with the birds' annual migration patterns. Then *V*. *cholerae* isolates should be sequenced and compared to establish the genetic characteristics of the strains selected by waterbirds at one location and deposited at another location all along the birds' migration route.

After the Haitian outbreak, comparative genetic analysis of *V*. *cholerae* strains from Haiti and Nepal suggested that the bacterium was transported from Nepal to Haiti by human mobility [22–24]. Nevertheless, this finding does not contradict the possibility that *V*. *cholerae* epidemic strains might result from waterbirds' dissemination. These 2 routes for overseas pandemic strains transportation might overlap and thus occur in parallel. We hope this review will inspire further studies regarding the understanding of the waterbirds' role in the global dissemination of *V*. *cholerae*.

## Unsolved questions and future research

Does *V*. *cholerae* colonize some waterbird species, or does the bacterium just pass through the birds' intestine after preying on fish or zooplankton? Feeding some waterbird species with green fluorescent protein (GFP) producing *V*. *cholerae*, should serve to answer this question. This should also be followed by observing the birds’ intestines to find out whether *V*. *cholerae* is attached to the intestines’ epithelial cells. If the bacteria colonize the intestine, are they transferred horizontally or vertically to their offspring? Does cholera toxin have some functions in waterbirds? Can we determine a model waterbird species that carries *V*. *cholerae*? Can we use this model to study and understand the role of *V*. *cholerae* pathogenic genes? Can we use this waterbird species model to monitor the dissemination of epidemic *V*. *cholerae* strains from one location to another and perhaps eventually to predict and even take measures to prevent cholera outbreaks?

## Supporting information

S1 Table A list of waterbird species from which *V. cholerae* were identified. The list specifies the phylogenetic position of each bird species. All waterbird species belong to the class Aves in the phylum Chordata.
(DOCX)
