# Supplementary material for: Accumulating evidence suggests that some waterbird species are potential vectors of Vibrio cholerae
Source: PLoS Pathog. 2019 Aug 22;15(8):e1007814. doi: 10.1371/journal.ppat.1007814 (PMC6706228; doi:10.1371/journal.ppat.1007814)
Supplement: S1 Table — The list specifies the phylogenetic position of each bird species. All waterbird species belong to the class Aves in the phylum Chordata. (DOCX) [file ppat.1007814.s001.docx]

**S1 Table**. **A list of waterbird species from which *V. cholerae* were identified.** The list specifies the phylogenetic position of each bird species. All the waterbird species belong to the class *Aves* in the phylum *Chordata*.

| **Order** | **Family** | **Genus** | **Species** |
| --- | --- | --- | --- |
| [*Anseriformes*](https://en.wikipedia.org/wiki/Anseriformes) | | | |
|  | [*Anatidae*](https://en.wikipedia.org/wiki/Anatidae) | [*Anas*](https://en.wikipedia.org/wiki/Anas) | *Anas acuta* (northern pintail) |
|  |  |  | *Anas carolinensis* (green-winged teal) |
|  |  |  | *Anas platyrhynchos* (mallard) |
|  |  |  | *Anas cyanoptera* (cinnamon teal) |
|  |  | [*Anser*](https://en.wikipedia.org/wiki/Anser_(genus)) | *Anser anser* (greylag goose) |
|  |  | [*Aythya*](https://en.wikipedia.org/wiki/Aythya) | *Aythya americana* (redhead) |
|  |  | [*Mareca*](https://en.wikipedia.org/wiki/Mareca) | *Mareca strepera* (gadwall) |
|  |  | *Spatula* | *Spatula discors* (blue-winged teal) |
| [*Charadriiformes*](https://en.wikipedia.org/wiki/Charadriiformes) | | | |
|  | [*Charadriidae*](https://en.wikipedia.org/wiki/Charadriidae) | [*Charadrius*](https://en.wikipedia.org/wiki/Charadrius) | *Charadrius vociferus* (killdeer) |
|  |  |  | *Charadrius wilsonia* (Wilson's Plover) |
|  | [*Laridae*](https://en.wikipedia.org/wiki/Laridae) | *Chroicocephalus* | *Chroicocephalus cirrocephalus* (grey-headed gull) |
|  |  | *Larus* | *Larus argentatus* (European herring gull) |
|  |  |  | *Larus californicus* (California gull) |
|  |  |  | *Larus delawarensis* (ring-billed gull) |
|  |  |  | *Larus* *dominicanus* (kelp gull) |
|  |  |  | *Larus marinus* (great black-backed gull) |
|  |  |  | *Larus ridibundus* (black-headed gull) |
|  |  |  | *Leucophaeus pipixcan* (Franklin's gull) |
|  |  | *Phaetusa* | *Phaetusa simplex* (large-billed Tern) |
|  |  | [*Thalasseus*](https://en.wikipedia.org/wiki/Thalasseus) | *Thalasseus acuflavidus* (Cabot’s tern) |
|  | *Scolopacidae* | [*Calidris*](https://en.wikipedia.org/wiki/Calidris) | *Calidris pusilla* (semipalmated sandpiper) |
|  |  | [*Phalaropus*](https://en.wikipedia.org/wiki/Phalarope) | *Phalaropus tricolor* (Wilson's phalarope) |
|  |  | [*Tringa*](https://en.wikipedia.org/wiki/Tringa) | *Tringa melanoleuca* (greater yellowlegs) |
|  | [*Jacanidae*](https://en.wikipedia.org/wiki/Jacanidae) | [*Jacana*](https://en.wikipedia.org/wiki/Jacana_(genus)) | *Jacana jacana* (wattled jacana) |
| [*Gruiformes*](https://en.wikipedia.org/wiki/Gruiformes) | | | |
|  | [*Rallidae*](https://en.wikipedia.org/wiki/Rallidae) | [*Fulica*](https://en.wikipedia.org/wiki/Fulica) | *Fulica americana* (American coot) |
| *Passeriformes* | | | |
|  | [*Corvidae*](https://en.wikipedia.org/wiki/Corvidae) | [*Corvus*](https://en.wikipedia.org/wiki/Corvus) | *Corvus frugilegus* (rook) |
| [*Pelecaniformes*](https://en.wikipedia.org/wiki/Pelecaniformes) | | | |
|  | [*Ardeidae*](https://en.wikipedia.org/wiki/Heron) | [*Ardea*](https://en.wikipedia.org/wiki/Ardea_(genus)) | *Ardea herodias* (great blue heron) |
|  |  | *Bubulcus* | *Bubulcus ibis* (cattle egret) |
|  |  | [*Egretta*](https://en.wikipedia.org/wiki/Egretta) | *Egretta thula* (snowy egret) |
|  |  |  | *Egretta garzetta* (little egret) |
|  |  | [*Nycticorax*](https://en.wikipedia.org/wiki/Nycticorax) | *Nycticorax nycticorax* (black-crowned night heron) |
|  | [*Pelecanidae*](https://en.wikipedia.org/wiki/Pelican) | [*Pelecanus*](https://en.wikipedia.org/wiki/Pelican) | *Pelecanus erythrorhynchos* (American white pelican) |
|  | [*Threskiornithidae*](https://en.wikipedia.org/wiki/Threskiornithidae) | [*Plegadis*](https://en.wikipedia.org/wiki/Plegadis) | *Plegadis chihi* (white-faced ibis) |
| [*Phoenicopteriformes*](https://en.wikipedia.org/wiki/Phoenicopteriformes) | | | |
|  | [*Phoenicopteridae*](https://en.wikipedia.org/wiki/Phoenicopteridae) | [*Phoenicopterus*](https://en.wikipedia.org/wiki/Flamingo) | *Phoenicopterus ruber* (American Flamingo) |
| *Procellariiformes* | | | |
|  | [*Diomedeidae*](https://en.wikipedia.org/wiki/Albatross) | [*Thalassarche*](https://en.wikipedia.org/wiki/Mollymawk) | *Thalassarche chlororhynchos* (Atlantic yellow-nosed albatross) |
|  | *Procellariidae* | *Puffinus* | *Puffinus puffinus* (Manx shearwater) |
| [*Suliformes*](https://en.wikipedia.org/wiki/Suliformes) | | | |
|  | [*Phalacrocoracidae*](https://en.wikipedia.org/wiki/Cormorant) | [*Phalacrocorax*](https://en.wikipedia.org/wiki/Cormorant) | *Phalacrocorax auritus* (double-crested cormorant) |
|  |  |  | *Phalacrocorax carbo* (great cormorant) |
|  | [*Sulidae*](https://en.wikipedia.org/wiki/Sulidae) | [*Sula*](https://en.wikipedia.org/wiki/Booby) | *Sula leucogaster* (brown booby) |
